# Numerical Evaluation of Human Body Near Field Exposure to a Vehicular Antenna for Military Applications

**DOI:** 10.3389/fpubh.2021.794564

**Published:** 2022-02-03

**Authors:** Micol Colella, Marianna Biscarini, Marco de Meis, Roberto Patrizi, Tino Ciallella, Daniele Ferrante, Alessandro De Gaetano, Marco Capuano, Giovanni Pellegrino, Emanuele Martini, Marta Cavagnaro, Francesca Apollonio, Micaela Liberti

**Affiliations:** ^1^Department of Information Engineering, Electronics and Telecommunications, Sapienza University of Rome, Rome, Italy; ^2^Larimart S.p.A., Rome, Italy; ^3^Centro Polifunzionale di Sperimentazione (CEPOLISPE), Rome, Italy

**Keywords:** computational dosimetry, vehicular antenna, human body model, RF exposure, safety

## Abstract

**Background:**

The use of electromagnetic (EM) technologies for military applications is gaining increasing interest to satisfy different operational needs, such as improving battlefield communications or jamming counterpart's signals. This is achieved by the use of high-power EM waves in several frequency bands (e.g., HF, VHF, and UHF). When considering military vehicles, several antennas are present in close proximity to the crew personnel, which are thus potentially exposed to high EM fields.

**Methods:**

A typical exposure scenario was reproduced numerically to evaluate the EM exposure of the human body in the presence of an HF vehicular antenna (2–30 MHz). The antenna was modeled as a monopole connected to a 3D polygonal structure representing the vehicle. Both the EM field levels in the absence and in the presence of the human body and also the specific absorption rate (SAR) values were calculated. The presence of the operator, partially standing outside the vehicle, was simulated with the virtual human body model Duke (Virtual Population, V.3). Several exposure scenarios were considered. The presence of a protective helmet was modeled as well.

**Results:**

In the area usually occupied by the personnel, E-field intensity radiated by the antenna can reach values above the limits settled by international safety guidelines. Nevertheless, local SAR values induced inside the human body reached a maximum value of 14 mW/kg, leading to whole-body averaged and 10-g averaged SAR values well below the corresponding limits.

**Conclusion:**

A complex and realistic near-field exposure scenario of the crew of a military vehicle was simulated. The obtained E-field values radiated in the free space by a HF vehicular antenna may reach values above the safety guidelines reference levels. Such values are not necessarily meaningful for the exposed subject. Indeed, SAR and E-field values induced inside the body remain well below safety limits.

## Introduction

Electromagnetic (EM) technologies for military applications are extensively used to satisfy a variety of operational requirements. Particularly, military vehicles are equipped with several antennas that work in a wide frequency range (e.g., high frequency–HF−3 to 30 MHz, very high frequency–VHF−30 to 300 MHz, and ultra-high frequency–UHF−300 to 3,000 MHz) and transmit high-power values for communications and jamming (1, 2). Due to their position in proximity to the manholes, vehicular antennas potentially expose the crew personnel to high-intensity EM fields (EMF) (3–5). International regulatory bodies, such as the International Commission on Nonionizing Radiation Protection (ICNIRP, mainly considered in Europe) or the IEEE Technical Committee 95 (IEEE-TC95, mainly considered in North America), aim to prevent health risks caused by EMF on both the general population and the workers, by setting limits in the exposure levels. For the workers, the limits are less conservative, since this category is considered to be exposed under controlled conditions (6). In this context, only IEEE-TC95 proposes a standard to specifically protect the personnel in a military workplace (7), whereas the ICNIRP does not distinguish for this category among the occupationally exposed individuals. Both IEEE and ICNIRP guidelines recommend two kinds of exposure limits. The first ones are based on dosimetric thresholds for established adverse health effects and are defined as *dosimetric reference limits* (DRLs) by the IEEE-TC95 (7) or *basic restrictions* (BRs) by the ICNIRP (6). Both limits can be expressed in terms of internal electric (E-)field strength and/or specific absorption rate, SAR (i.e., the power absorbed per unit mass), depending on the operating frequency (6, 7). In the frequency range between 1 and 30 MHz, IEEE DRLs are expressed in terms of SAR and impose a threshold value equal to 0.4 W/kg as averaged over the whole body (SAR_wb_), and 10 W/kg peak averaged over any 10 g of tissue (SAR_10Avg_), with the exception of extremities and pinnae where the limit is set at 20 W/kg (7). In the same frequency range, ICNIRP BRs are very close to those settled by IEEE, for exposure durations greater than 6 min. The second settled limits are the IEEE *exposure reference levels* (ERLs) and the ICNIRP *reference levels* (RLs), which represent a more practical method to determine compliance with the guidelines (6, 7). The IEEE ERLs are derived from the DRLs and are given as RMS quantities spatially averaged over an area equivalent to the vertical cross-section of the human body (projected area). In the frequency range between 1 MHz and 30 MHz, the limit is calculated as 1842/f_M_ (V/m), where f_M_ is the frequency in MHz (7). Such ERL level is valid in the so-called Zone 1, where only informed and instructed personnel is allowed (7). The ICNIRP guidelines do not include such differentiation in the exposure location (6), but they introduce differences in the settled limits related to the duration of the exposure. ICNIRP RLs represent the unperturbed electric field or power density values, calculated in the area in which the human body can be located but in its absence. They are derived from the BRs (6). In the 2020 ICNIRP guidelines, RLs for exposures longer than 30 min are given as equal to 660/f${}_{\text{M}}^{0.7}$ (RMS, whole-body averaged unperturbed values), whereas shorter exposures allow more relaxed values. In the frame of the European legislation, the Directive 2013/35/UE referred to the previous ICNIRP 2010 guidelines (8), to derive the so-called *exposure level values* and the *action levels*, that establish the minimum health and safety requirements regarding the exposure of workers to the risks arising from EMF exposure (9, 10). In particular, the exposure level values are derived from ICNIRP 2010 BRs, whereas action values are derived from the RLs, with the important modification that action levels are considered as the maximum field value in the area occupied by the human body, rather than whole-body volume averaged values. Finally, in the context of personnel protection from EMF, each European Member State can adopt the EU directive or implement equivalent or more specific protection systems, such as the North Atlantic Treaty Organization (NATO) international standard agreement (STANAG 2345 (11)) that specifically protects armed forces. The rationale proposed by both IEEE and ICNIRP and adopted by the European directive, of providing two exposure limits, allows for a first and rapid verification of compliance with the guidelines by a direct measure of external quantities, such as power density or field strength. In the case of exceeding the RLs, further investigation must be carried out to verify that the BRs are respected (12). Within this framework, computational dosimetry becomes a fundamental tool as it gives the possibility to realistically reproduce an exposure scenario and study the EM quantities induced inside the human body. Different numerical studies have been conducted to evaluate exposure levels around and inside standard (3, 13, 14), or military (1, 2, 15) vehicles equipped with radiofrequency (RF) antennas. Nevertheless, they either account for the presence of the human body with homogeneous virtual phantoms (13, 14) or do not account for it at all (1, 2, 15). More recently, a study took into account heterogeneous body models (3). Particularly, the aim of the study conducted by Guellab and Wu (3) was to develop a four-pole Debye model of the dielectric properties of each tissue, to take into account the frequency dependence. To validate such model, they simulated the exposure of the crew placed inside a military vehicle equipped with a UHF (100 MHz−1 GHz) high-power antenna. Nevertheless, as pointed out by Sobiech and colleagues (4), inside the vehicle, the EM hazard is reduced because of the extended distance between the antennas and personnel, and thanks to the shielding effect of the vehicle metallic structure. Conversely, in the area outside the vehicle and near the EMF source, the maximum intensity can exceed the guideline limits. To this regard, current literature does not provide a deep analysis of the personnel exposure in the proximity of a high-power radiating antenna, as would happen in correspondence of the turret manhole. Furthermore, there is a lack of information about the effect of wearing personal protective equipment (i.e., a helmet) or cabled instrumentation for communication. Thus, the aim of this study was to investigate realistic scenarios of an operator standing partially outside the vehicle, close to a vehicular antenna working in the HF frequency range (2–30 MHz), to deepen knowledge in terms of both induced electric field and SAR inside the body. Furthermore, conditions such as the use of a helmet equipped with a headset were investigated to verify whether it exposes the head of the operator to potentially high intensities. To the best of the authors' knowledge, this is the first time that a dosimetric analysis is performed in such a realistic scenario with safety purposes.

## Materials and Methods

### Modeling the Vehicular Antenna

**Figure 1:**
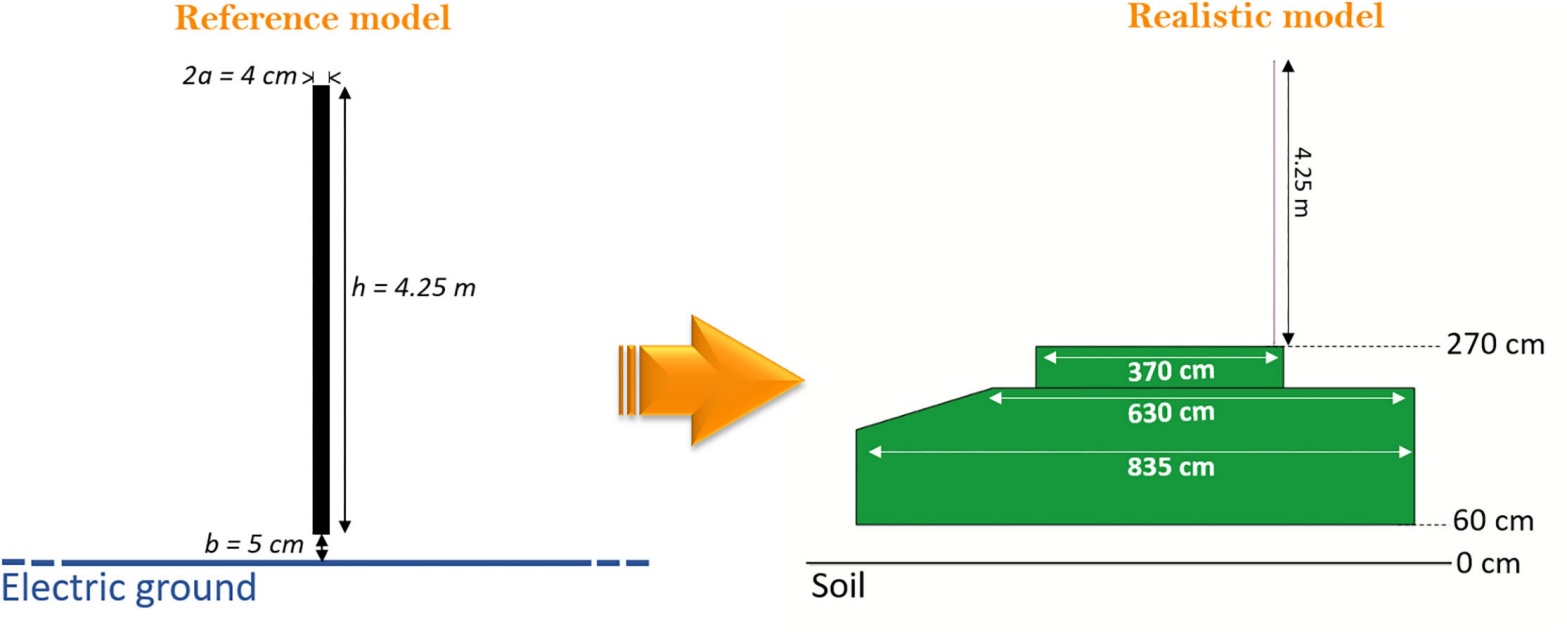
Numerical model of the antenna. In the reference model, the monopole (4.25 m high, diameter, *2a*, of 4 cm and feeding gap, *b*, of 5 cm) is placed over an ideal infinite electric ground. In the realistic model, the monopole is mounted over a simplified 3D model of the vehicle, reproducing typical dimensions. A 60cm gap is considered between the bottom of the vehicle and the soil to simulate the presence of the tires.

**Figure 2:**
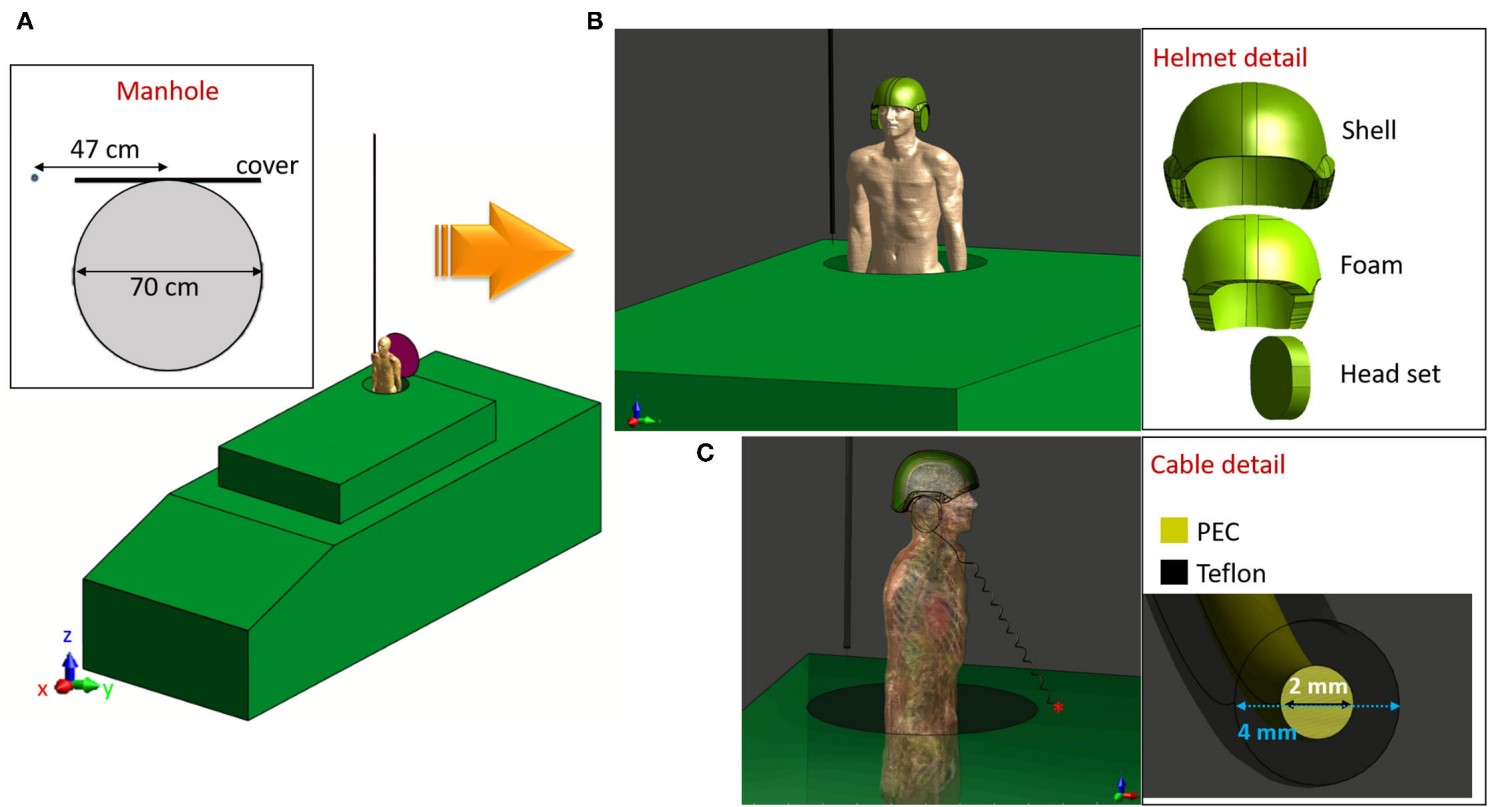
Military personnel exposure scenario: **(A)** Unequipped operator standing partially outside the manhole, inset: detail of manhole position and dimensions. **(B)** Equipped operator wearing the protective helmet, inset: detail of the helmet composition. **(C)** Equipped operator wearing the protective helmet and the cabled headset, connected to the vehicle through a 10μF capacitor, inset: detail of the connecting cable.

HF vehicular antennas operate in the frequency range between 2 and 30 MHz. Usually, they are monopoles with the vehicle metal sheet that makes the monopole ground. In this study, the antenna was made by a conductor 4.25 m long. The radius *a* and the feeding gap *b* (Figure 1) were dimensioned based on antenna theory (16), resulting in 2 and 5 cm, respectively. Further details on the model can be found in the Supplementary Material. The study was performed at the HF central frequency of 16 MHz with 25 W of input power. Ideally, a monopole should be connected to an infinite (>> λ) ground plane, as shown in Figure 1. Nevertheless, in such applications, the antenna is placed on the roof of the vehicle, which is characterized by finite dimensions. Therefore, to respect a typical military exposure scenario, the infinite electric ground was replaced with a 3D simplified reproduction of a military vehicle, respecting the dimensions of all the critical sections, as shown in Figure 1. In the vehicle model, the base has a size of 835 x 300 cm, and the middle part (630 x 280 cm) is tapered and connects the base to the turret (370 x 220 cm). The manhole on the turret and its door were considered as well. The manhole has a diameter of 70 cm and is placed at about 50 cm from the antenna (Figure 2, manhole inset). The vehicle, the monopole, and the manhole door were assigned to a perfectly conductive (PEC) material. To investigate a possible coupling between the monopole and the metallic door, the latter was simulated open. The soil was simulated as a plane of PEC, and a 60 cm gap was considered to mimic the tires, as shown in Figure 1.

### Modeling the Exposure Scenario

To take into account the presence of the operator, the whole-body model Duke (standard adult men, aged 34 years, 1.77 cm tall and 70 kg) weight of the Virtual Population (ViP v.3) was used (17). The Duke model was located in correspondence of the manhole, with 70 cm of the body (i.e., the trunk) standing outside the vehicle, as shown in Figure 2A. The level of detail used in modeling the exposure scenario was progressively increased by adding a protective helmet and a cabled headset. The helmet is made of a ballistic shell, a liner containing a foam padding, and the headset case (Figure 2B, helmet inset). The model of the intercom cable passes through the headset case and around the nape to fall along the operator's body in a helix of a length of 120 cm (Figure 2C). The cable is made of a Teflon jacket of 4 mm diameter and a copper wire of 2 mm diameter (Figure 2C, cable inset), and it is connected to the external surface of the vehicle (functioning as the ground), by a 10 μF capacitor. As a whole, four exposure scenarios were simulated:

- *Exposure scenario a*: vehicle with open manhole,
- *Exposure scenario b*: vehicle with the operator partially outside the manhole,
- *Exposure scenario c*: vehicle with the operator partially outside the manhole, wearing the protective helmet,
- *Exposure scenario d*: vehicle with the operator partially outside the manhole, wearing the protective helmet, and the cabled headset.

Additionally, exposure scenario d was further investigated in the absence of the human body, to understand the effect of the cabled headset on the E-field intensity radiated in air. In Scenarios c and d, polyamide was assigned to the shell and the foam padding of the helmet, nylon to the headset case. The copper wire of the cable was modeled as PEC, and the cable's insulating jacket was modeled as Teflon. The dielectric properties assigned to each material at 16 MHz can be found in Table 1. The physical properties (i.e., electrical conductivity, electrical permittivity, and mass density) of each of the 305 anatomical elements of the Duke model were assigned from the IT'IS data base (18) at the working frequency. In all the scenarios, the reference system was chosen as oriented with the Z-axis along the length of the antenna, and the XY plane parallel to the surface of the vehicle, with the length of the vehicle along the X-axis and the width along the Y-axis, as shown in Figure 2.

**Table 1:** Dielectric properties of the materials considered to model the helmet and the cable.

**Material**	**Conductivity**	**Relative permittivity**
Polyamide	6.6 *times* 10^−6^ S/m	3.5
Nylon	0.0057 S/m	2.84
Teflon	4.62*times* 10^−4^ S/m	1

### Electromagnetic Simulations

The exposure scenarios were modeled and simulated within the simulation software Sim4Life (v.5, Zurich MedTech, Zurich). The EM problem was solved using the finite difference time domain (FDTD) method (19), considering a 16 MHz sinusoidal source feeding the monopole. To ensure free propagation of the E-field outside the simulation domain, absorbing boundary conditions with perfectly matched layer (PML) were assigned to the lateral and superior walls, whereas a PEC condition was assigned to the inferior wall to simulate the presence of the reflecting soil. A non-uniform grid was applied to each scenario. The vehicle was discretized with a 5-cm isotropic grid, whereas an anisotropic grid (2 mm x 2 mm x 3 cm) was considered for the monopole antenna. Additionally, Duke's head, the helmet, and the cable were discretized with an isotropic 2-mm resolution, whereas 2.5 mm was used on Duke's trunk. The overall number of cells in the simulation space was 13 MCells for *scenario a*, 66 MCells for *scenario b*, 68 MCells for *scenario c*, and 98 MCells for *scenario d*. The results of the simulations were analyzed focusing on the induced E-field, and also on the whole-body averaged SAR (SAR_wb_), for global exposure, and on the peak of the SAR averaged over 10 g of tissue in the head (SAR_10Avg_), for local exposure. Following the safety guidelines rationale, that is, to always look for the worst-case exposure, the calculated field values are analyzed with respect to the lowest limit among those settled in the different guidelines. Accordingly, 86.3 V/m is taken in the following as the threshold for RLs, derived from the 61 V/m (RMS), considered as the E-field maximum value in the area where the body can be located (8, 9). As described in the Introduction section, in the cases in which the unperturbed field values are above the thresholds, SAR values should be considered. In this latter case, all reported guidelines set the same limits, that is, 0.4 W/kg averaged over the whole body, or 10 W/kg peak SAR averaged over 10 g of tissue (6, 7, 9).

## Results

### Exposure Scenario *a*: Vehicle With Open Manhole

**Figure 3:**
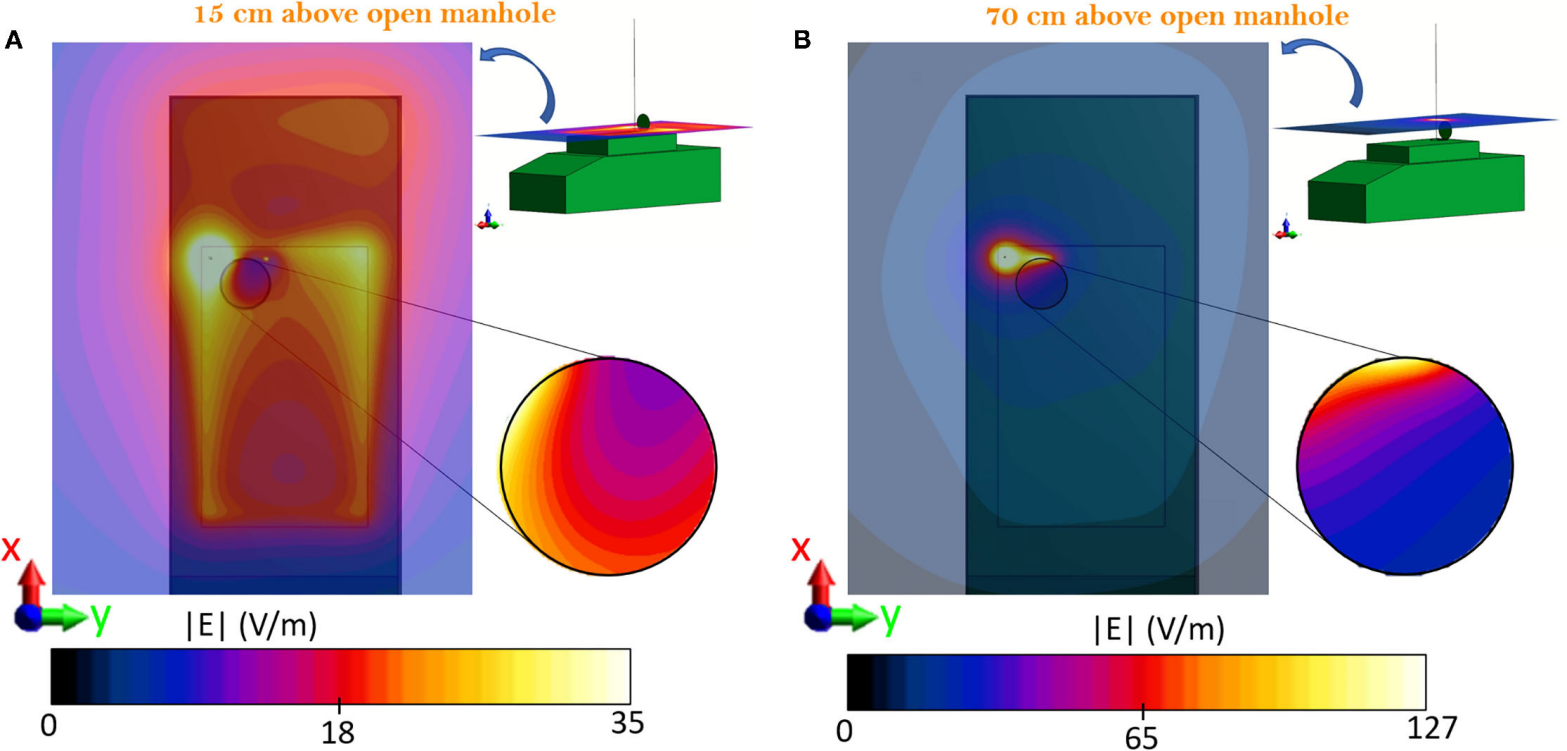
Top view of the vehicle and distribution map of the E-field radiated by the antenna model fed with 25 W at 16 MHz. **(A)** Distribution at 15 cm above the manhole, circular inset: detail of the manhole area. **(B)** Distribution at 70 cm above the manhole, circular inset: detail of the manhole area. The vehicle and manhole boundaries are shown by the light black lines.

The E-field generated by the monopole placed over the simplified model of the vehicle was analyzed in the absence of the human body, with particular attention to the values estimated in the proximity of the manhole. Assuming that the space above the manhole would be usually occupied by the trunk of an average size man, it was of interest to evaluate the E-field distribution in areas located at different heights from the surface of the manhole, that is, h_1_ = 15 cm and h_2_ = 70 cm, which approximately correspond to the location of the waist and of the head, respectively. The E-field distributions at the two heights h_1_ and h_2_ are reported in Figure 3. In both planes, a coupling between the monopole and the metallic manhole door and the influence of the open manhole are visible. At h_1_ = 15 cm, a decrease in the E-field intensity is caused by the presence of the open manhole. In particular, within a 10 cm radius around the antenna, levels above 100 V/m were estimated, that decrease to values between 35 and 11 V/m over the manhole (Figure 3A). A hotspot of 40 V/m was found at the farther extremity of the manhole door, and, as expected, an increase in the field intensity occurred in correspondence of the sharp corner of the turret of the vehicle, as well. Higher intensity values were found at 70 cm (Figure 3B), where the presence of the metallic door deforms the E-field distribution causing peaks above 100 V/m close to the door area. Inside the area directly above the manhole, the non-uniform distribution of the E-field was characterized by an intensity that ranged from 127 to 20 V/m, with an average value of 37.45 V/m. To conclude, in the exposure scenario herein depicted, local E-field intensity values may be above the considered RL (9), particularly at 70 cm. Therefore, further dosimetric analysis is required to ensure compliance with the SAR BRs.

### Exposure Scenario *b*: Vehicle With Open Manhole in the Presence of the Operator

**Figure 4:**
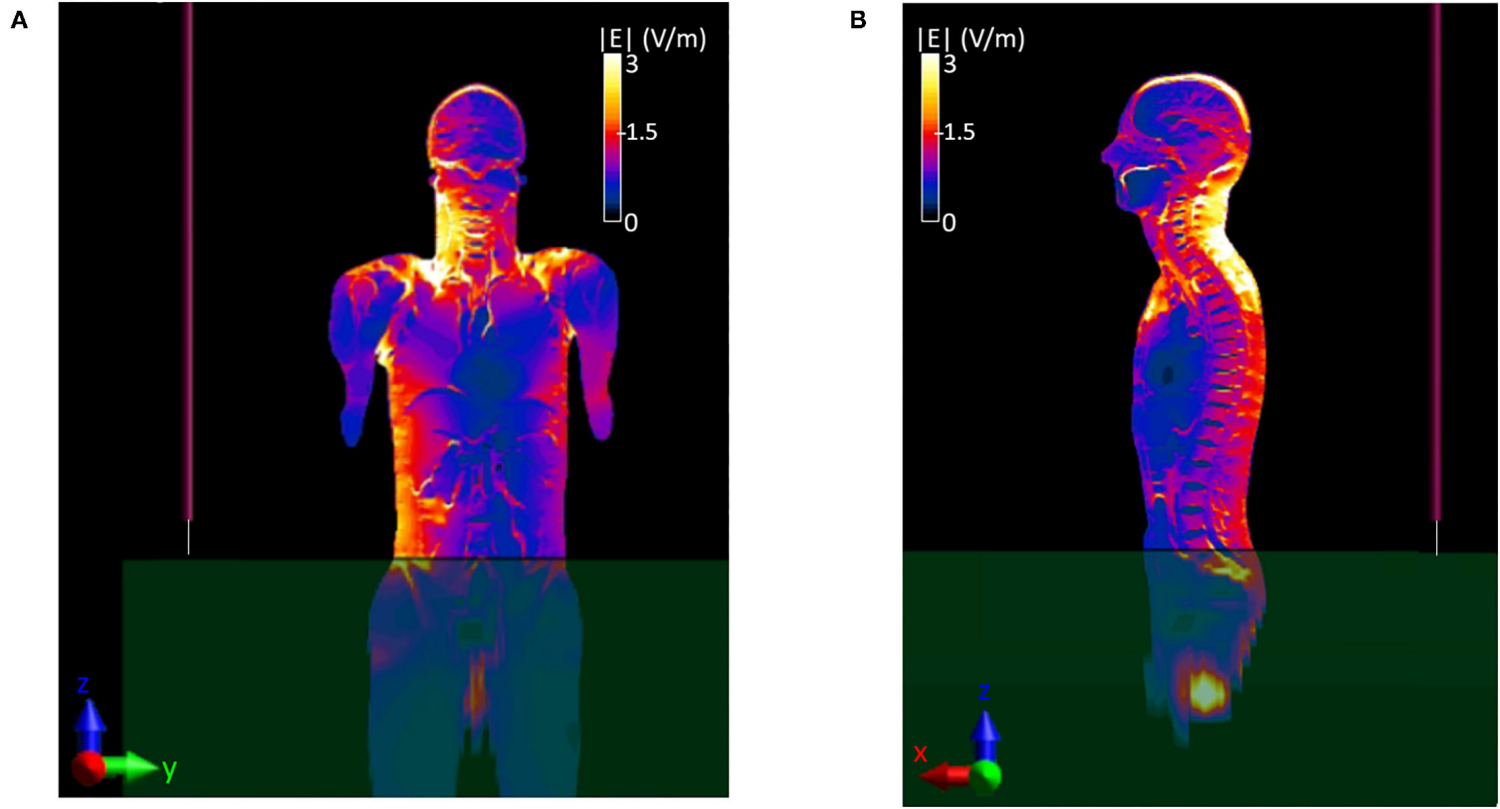
Distribution of the E-field induced inside the virtual body model Duke. **(A)** Coronal or frontal plane-YZ, **(B)** sagittal or lateral plane-XZ.

**Figure 5:**
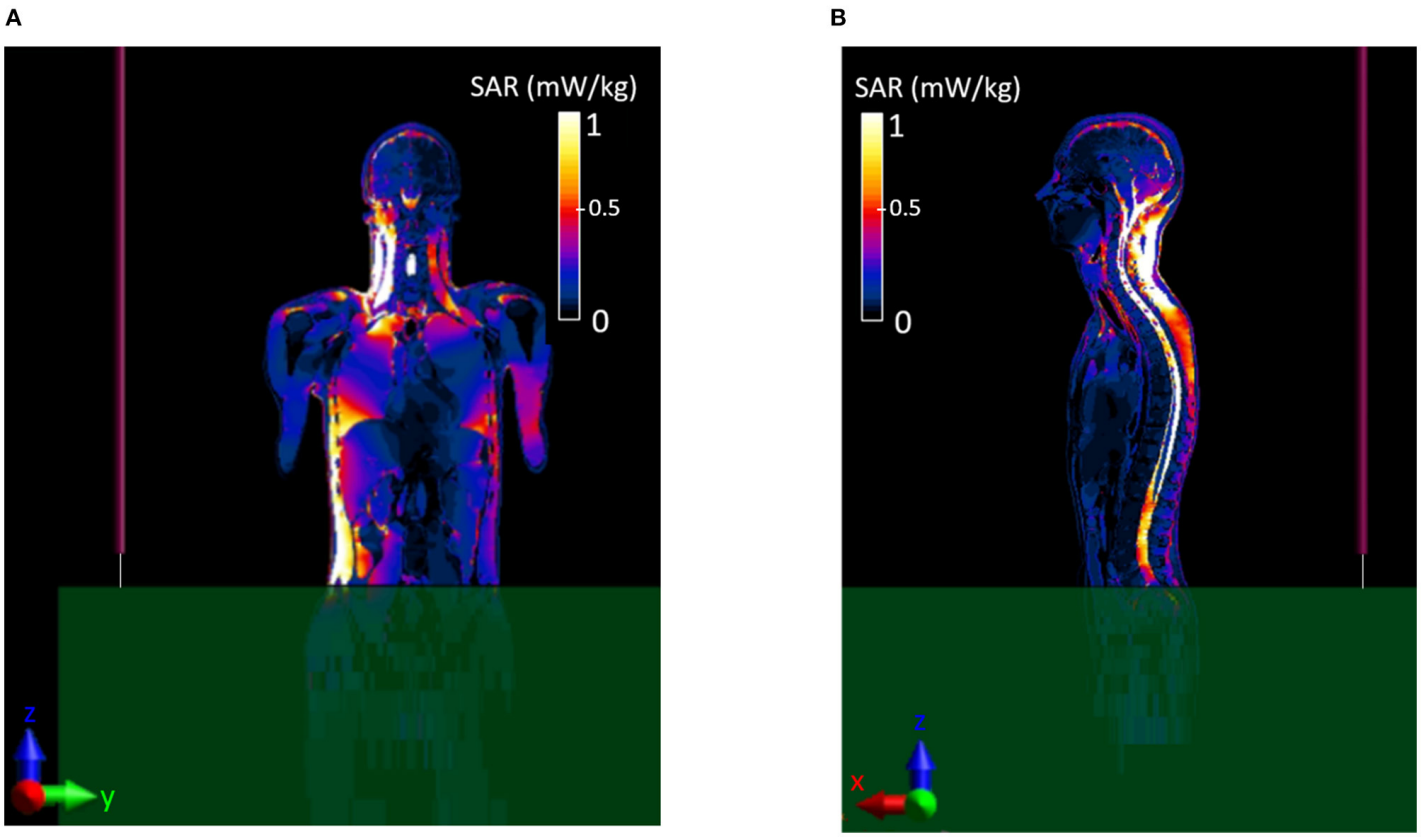
Local distribution of the induced SAR inside the virtual body model Duke. **(A)** Coronal or frontal plane-YZ, **(B)** sagittal or lateral plane-XZ.

**Figure 6:**
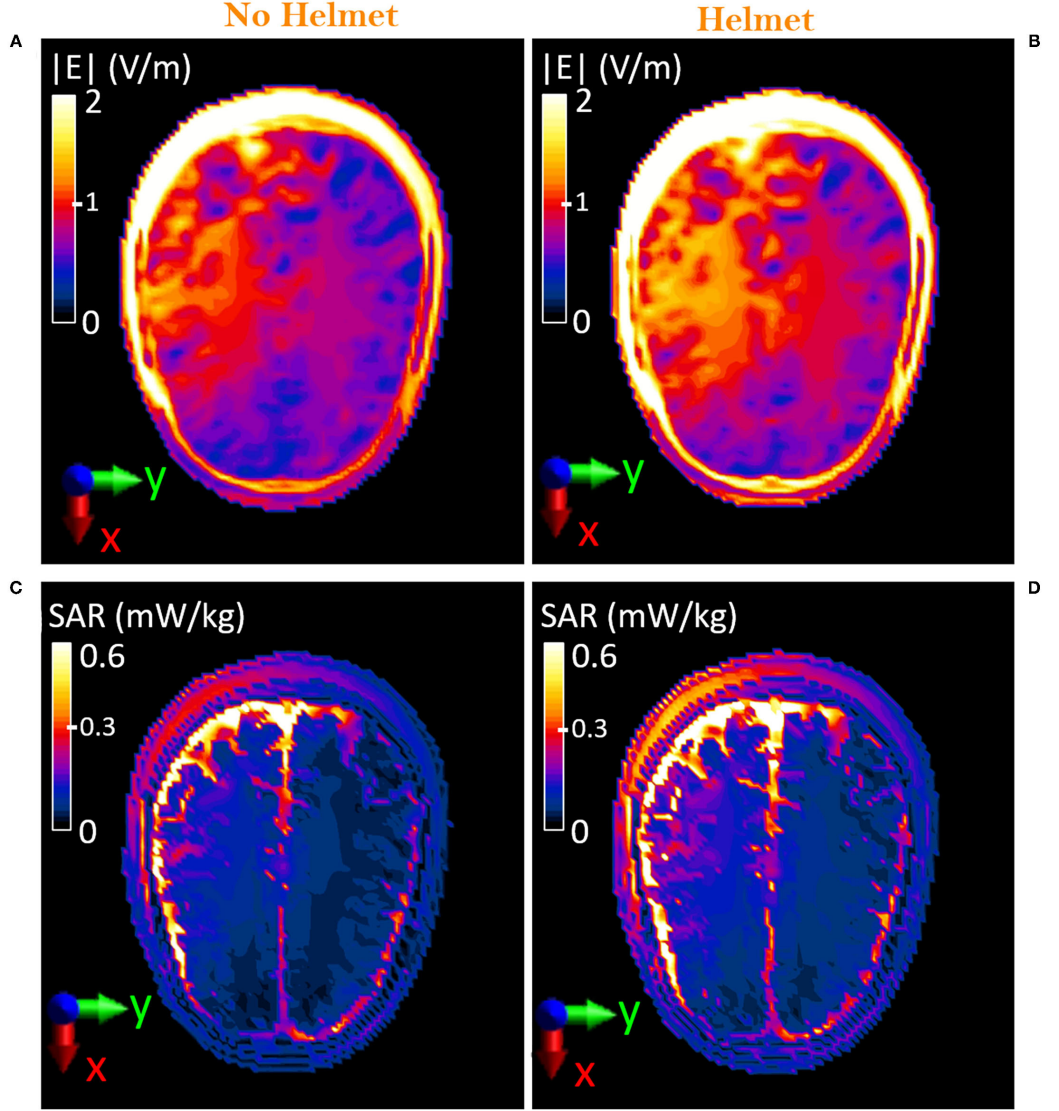
Comparison on the axial plane, XY, between unequipped and equipped military operator, (XY plane): **(A,B)** Distribution of the E-field induced inside the head of the virtual body model Duke at 70 cm from the manhole. **(C,D)** Distribution of the local SAR induced inside the head of the virtual body model Duke at 70 cm from the manhole.

Figure 4 shows the E-field distribution that was induced inside the Duke model over two planes, YZ (Figure 4A) e XZ (Figure 4B), at the center of the body. The right side of the body is majorly interested by the exposure, as it is located in the proximity of the monopole. A hotspot of 12.3 V/m is induced in the valley between neck and shoulders (Figure 4A) and in the cervical area (Figure 4B), whereas values below 3 V/m are induced in the rest of the body. To evaluate the impact of such values, the corresponding induced SAR values were evaluated as well. The local SAR distributions over the same two planes are reported in Figure 5. Location of the hotspot is maintained with a peak SAR value of 14.7 mW/kg. Given the previously shown high E-field intensity values radiated in free space at 70 cm from the manhole, the values that were induced inside the Duke model at the same location, which corresponded to the center of the head, were evaluated (Figure 6A). Results showed that values below 2.8 V/m were induced inside the cerebral tissues and lead to a local SAR below 0.6 mW/kg (Figure 6C). To ensure compliance with the guidelines, the whole-body averaged SAR (SAR_wb_) and the peak SAR averaged over 10 g of tissue (SAR_10Avg_) in the head were computed and resulted to be 0.2 and 3.2 mW/kg, respectively. These values were well below the limits reported in the guidelines, as shown in Table 2. A different version of scenario b, with the operator's body placed towards the edge of the manhole and the arm bent over the turret (i.e., scenario b1), was investigated as well, and results can be found in the Supplementary Material.

**Table 2:** Comparison between the estimated SAR values in the three exposure scenarios that include the operator model and guidelines limits.

	**Estimated value**			**Guidelines limit (6, 7, 9)**
	***Scenario b:* vehicle with operator**	***Scenario c:* vehicle with operator and helmet**	***Scenario d:* vehicle with operator, helmet and cabled headset**	
SAR_wb_	0.20 ×10^−3^ W/kg	0.21 ×10^−3^ W/kg	0.21 ×10^−3^ W/kg	0.4 W/kg
SAR_10Avg_	3.2 ×10^−3^ W/kg	3.7 ×10^−3^ W/kg	13.97 ×10^−3^ W/kg	10 W/kg

### Exposure Scenario *c*: Operator Equipped With the Protective Helmet

The presence of the protective helmet slightly influences the E-field that was induced inside the body of the Duke model. Particularly, changes would be mainly expected at the level of the head, where the helmet is located. Figures 6A,B show a comparison of the E-field distribution over the axial (XY) plane at the forehead (70 cm above the manhole) in the case of Duke without and with the protective helmet, respectively. The helmet causes a 10% increment in the induced E-field levels. Nevertheless, values were kept below 3.1 V/m in the cutaneous tissues and below 1.9 V/m in the cerebral tissues, such as the gray and the white matter. As for exposure scenario b, the induced SAR levels were investigated. Local SAR on the forehead plane is shown in Figures 6C,D. When the helmet is introduced, an increment of the SAR levels with respect to the levels obtained in the absence of the helmet is visible. The maximum local SAR, estimated as the 99.9th percentile of the SAR distribution inside the head (20), increases from 2.9 mW/kg (in Figure 6C) to 4.5 mW/kg (in Figure 6D). To evaluate compliance with the guidelines, the computed peak SAR averaged on 10 g of head tissues is 3.7 mW/kg, that is, 15% higher than the value that would be induced without the helmet but still well below the 10 W/kg limit. Conversely, the SAR_wb_ remains unchanged (Table 2).

### Exposure Scenario *d*: Operator Equipped With the Protective Helmet and Cabled Headset

**Figure 7:**
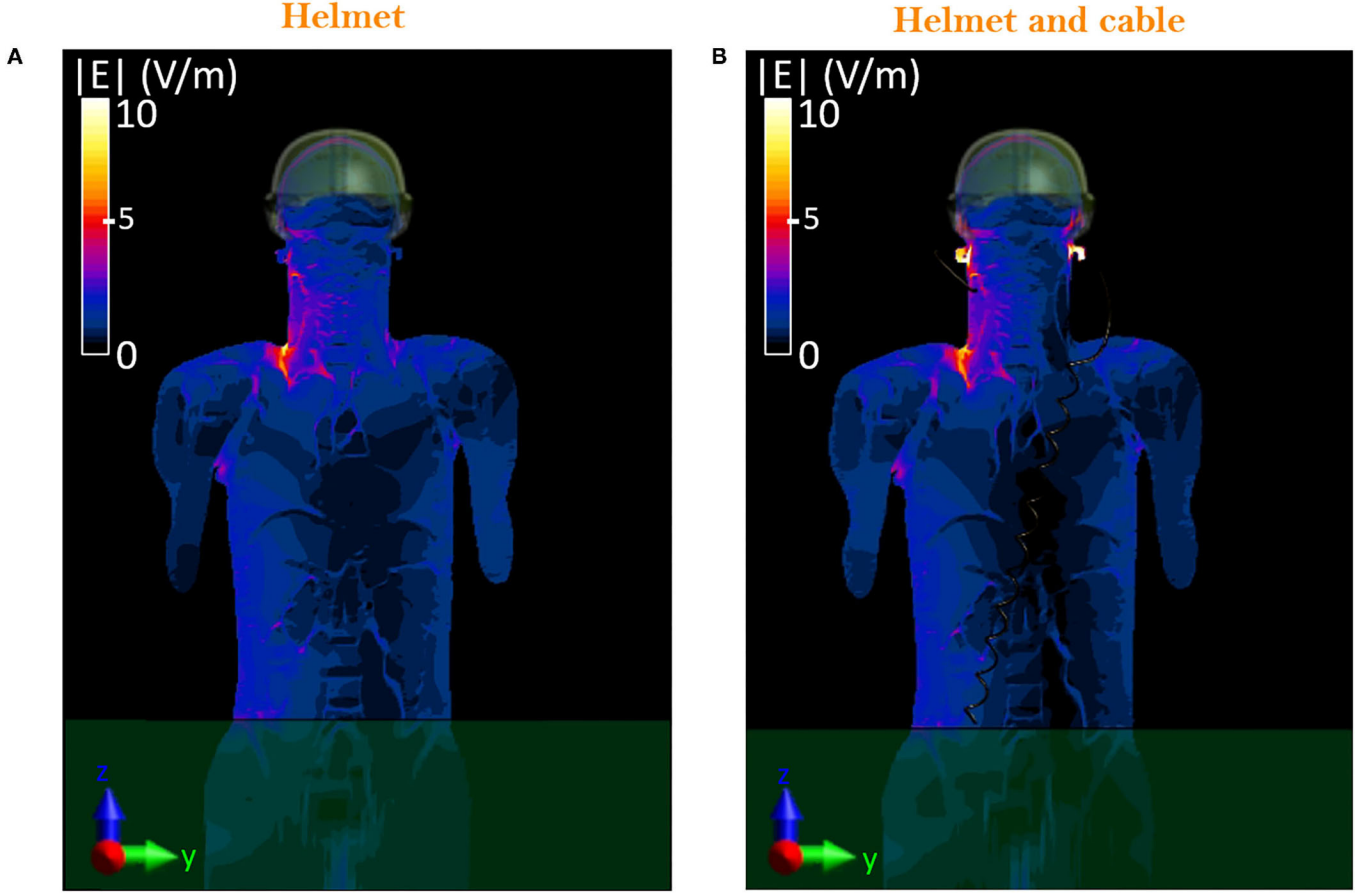
Distribution of the E-field induced inside the head of the virtual body model: comparison on the frontal plane, YZ, between equipped military operator wearing the sole helmet **(A)** and the helmet with the cabled headset **(B)**.

**Figure 8:**
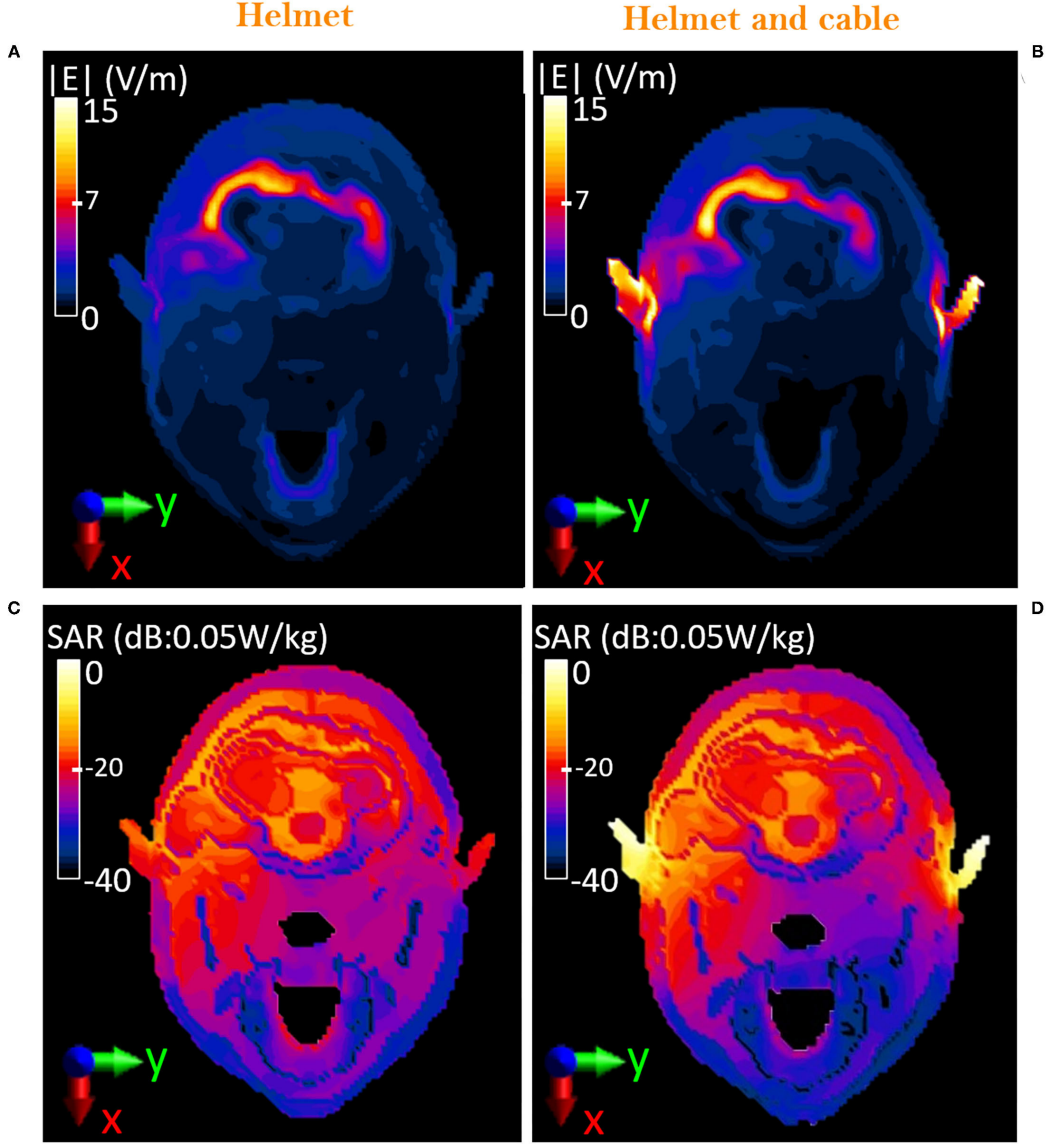
Comparison on the axial plane, XY, between equipped military operator wearing the sole helmet and the helmet with the cabled headset. Distribution of the E-field induced inside the head of the virtual body model Duke at 65 cm from the manhole **(A,B)**. Distribution of the local SAR induced inside the head of the virtual body model Duke at 65 cm from the manhole **(C,D)**. Color bar for SAR is reported in logarithmic scale, considering 0.05 W/kg as the reference value (i.e., 0 dB = 0.05 W/kg).

**Figure 9:**
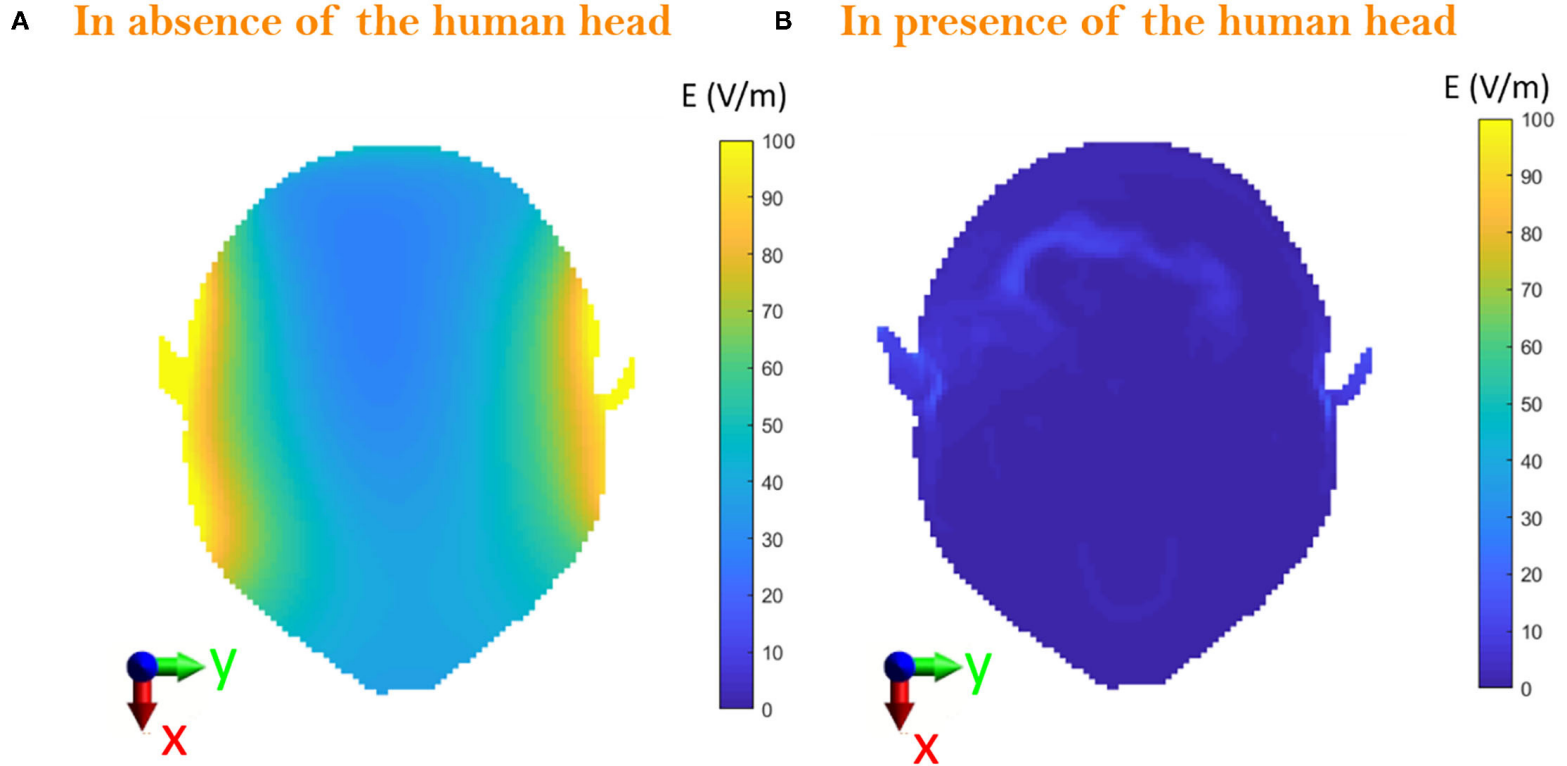
Comparison on the axial plane, XY, between **(A)** the E-field radiated in the space surrounded by the helmet in absence of human head and **(B)** the E-field induced inside the head with the operator wearing the helmet at 65 cm from the manhole.

Figure 7 shows the induced E-field inside the whole-body Duke model in the two cases of the operator wearing the sole protective helmet (Figure 7A) and the protective helmet with the cabled headset (Figure 7B). When in the presence of the cable, the induced E-field is below 3 V/m everywhere except for the areas of the ears where the presence of the cable causes a peak of 49.8 V/m. Since the cable runs from left to right behind the neck, the effect of the connected headset can be seen at the level of the nape as well (i.e., 65 cm above the manhole), as shown comparing Figure 8A with Figure 8B, where the peak intensity, again estimated as the 99.9th percentile, increases from 12.9 to 15.1 V/m. Nevertheless, despite the cable is close to the area of the chest and abdomen, the E-field induced in those areas is not influenced by its presence. In Figures 8C,D, the local induced SAR is shown for the two cases. The main difference is again on the ears, where the local SAR increases from 1.5 mW/kg (Figure 8C) to 40 mW/kg (Figure 8D), whereas the SAR_wb_ remains unchanged (Table 2). To investigate whether the hotspot on the ears would cause a non-compliance with the BRs, the peak head SAR_10Avg_ was computed and resulted equal to 13.97 mW/kg, well below the recommended 10 W/kg (Table 2). To correlate these results with the corresponding RLs that could be found in air, *exposure scenario d* was investigated in the absence of the Duke model. Results were compared with those obtained in the presence of the head inside the the helmet and were analyzed over the plane at 65 cm from the manhole (Figure 9). When considering the connected cable without the presence of the head, the mean value inside the helmet space is 46.7 V/m, whereas the peak value reaches 132 V/m. Higher intensity values are focused in correspondence of the right and left headset (Figure 9A), which means that the cable acts as a receiving antenna, which concentrates the E-field lines along its length. When in the presence of the head tissues (Figure 9B), the induced E-field was up to 49.8 V/m, with a mean value of 2 V/m.

## Discussion and Conclusions

In this study, a realistic military scenario was simulated considering a highly detailed virtual human body model standing partially outside a vehicle. The vehicle was modeled as a low complexity replica of a real one. The RF antenna was modeled as a monopole placed on the roof of the vehicle. Numerical simulations were performed at 16 MHz, which is the central frequency of the working range 2–30 MHz, and considering an input power of 25 W. Four different conditions were investigated. The E-field levels in the free space without the human body were evaluated first (*scenario a*). Following, the exposure scenario in the presence of the operator was simulated (*scenario b*), and the level of detail in the exposure scenario was progressively increased by adding the protective helmet (*scenario c*) and a pair of cabled headsets (*scenario d*). In *scenario a*, it was estimated that areas above the manhole were mostly compliant with the limits at 16 MHz (i.e., 61 V/m rms or 86.3 V/m magnitude) (9), except for a small area where the local E-field intensity reached 127 V/m. To assess whether such intensity values would induce SAR and E-field values inside the body that exceed the BRs, the presence of the military operator, partially standing outside the vehicle, through a manhole, in close proximity to the radiating monopole, was considered. As a first step, the unequipped operator was modeled (*scenario b)* similarly to what was done in other dosimetric studies (3, 21). The induced E-field inside the body was estimated to be up to 12 V/m, leading to SAR_wb_ and a peak SAR_10Avg_ in the order of 10^−3^ mW/kg, well below the limits of 0.4 and 10 W/kg, respectively (6, 7, 9), thus finding that noncompliant E-field induced compliant SAR_wb_ and peak SAR_10Avg_, as also found by Alcaras and colleagues in 2017, under similar exposure condition (21). To take into account a more realistic scenario, the equipment usually worn by the personnel was modeled. Specifically, a protective helmet with a cabled headset was added to the FDTD simulations, to investigate whether these elements could be detrimental due to a possible direct coupling with the antenna. Such modeling details were progressively added, by running two sets of simulations, one with the sole helmet and one with the helmet plus the cabled headset. To the best of our knowledge, this aspect has never been considered in the previous dosimetric studies.

When the man is modeled with the protective helmet alone (*scenario c*), the induced E-field inside the head is 10% higher than the one obtained in case b (i.e., man without helmet) but always below 3.1 V/m and inducing a peak head SAR_10Avg_ of 3.7 mW/kg. When the cabled headset is included in the simulation (*scenario d*), a local increase of the E-field and induced SAR at the level of ears is observed, although with values still well below the guideline's limits. The presence of the helmet and the cabled headset was investigated in the absence of the operator model, as well, for a better understanding of the hotspot occurring at the level of the ears. The local increase of the E-field was found in the area surrounding the right and left elements of the headset. This allowed to conclude that such local increase of the E-field is caused by the induction of currents inside the cable, which, in turns, act focusing the EM field, rather than being an effect of the ears' shape and dielectric properties. Nevertheless, SAR and E-field values that were induced inside the body in such condition remained well below the BR's limits, with a peak head SAR_10Avg_ of 14 mW/kg. With these findings, giving the linear regime of the problem studied, even when considering the worst-case exposure scenario (scenario d), the respect of the BRs is still guaranteed even if the antenna is fed in more extreme conditions, such as 125 W (2). For instance, under these circumstances, the peak SAR_10Avg_ would be 69.85 mW/kg, still below the ICNIRP limit of 10 W/kg. In conclusion, a computational model that represents a good compromise between accuracy and efficiency was herein proposed to perform dosimetric evaluation for safety assessment of the exposure of military crew in high-power near-field conditions in the HF range. A realistic exposure scenario was simulated, in which the radiated electric field reached values that could be above the guidelines RLs and that coupled with the instrumentation, such as wearable communication systems. Nevertheless, it was shown that the E-field and SAR that were induced inside the operator's body located in the proximity of the radiating antenna would still respect the guideline limits for occupational exposure at the selected frequency.

## Data Availability Statement

The original contributions presented in the study are included in the article/Supplementary Material, further inquiries can be directed to the corresponding author/s.

## Author Contributions

MB and TC contributed in project administration. MB, MCav, FA, and ML supervised the work. MCo, MB, MCav, FA, and ML performed conceptualization, methodology, investigation, and wrote the original draft. MCo carried out formal analysis. MM, RP, TC, DF, AD, MCap, GP, and EM provided the resources, involved in reviewing, and editing. All authors contributed to the article and approved the submitted version.

## Conflict of Interest

MM, RP, and TC are employees at Larimart S.p.A. DF, AD, MCap, GP, and EM are employees at CEPOLISPE. Larimart and Cepolispe provided information about vehicle and antenna dimensions. This work was carried out in the frame of the HEPROSYS project funded by Lariamrt S.p.A. The remaining authors declare that the research was conducted in the absence of any commercial or financial relationships that could be construed as a potential conflict of interest.

## Publisher's Note

All claims expressed in this article are solely those of the authors and do not necessarily represent those of their affiliated organizations, or those of the publisher, the editors and the reviewers. Any product that may be evaluated in this article, or claim that may be made by its manufacturer, is not guaranteed or endorsed by the publisher.
