# Supplementary material for: Numerical Evaluation of Human Body Near Field Exposure to a Vehicular Antenna for Military Applications
Source: Front Public Health. 2022 Feb 3;9:794564. doi: 10.3389/fpubh.2021.794564 (PMC8851057; doi:10.3389/fpubh.2021.794564)
Supplement: Supplementary file 1 [file Data_Sheet_1.docx]

Supplementary Material

# Verification of the FDTD model

The FDTD model of the monopole built and simulated inside the EM-simulation software Sim4Life (v.5, Zurich MedTech, AG) was compared with an identical model solved within the Antenna ToolBox (Matlab R2020, MathWorks Inc.), that applies the methods of moments (MoM). Since the Antenna ToolBox is not able to manage 3D structures to act as the ground for the modeled antenna, in both the software packages planar grounds were considered. Two grounds of finite dimensions were considered:

- 20 m x 20 m (≈ λ, Supp. Fig. 1 A-B)
- 3 m x 8.35 m (equal to the main dimension of the vehicle, Supp. Fig. 1 A-B)


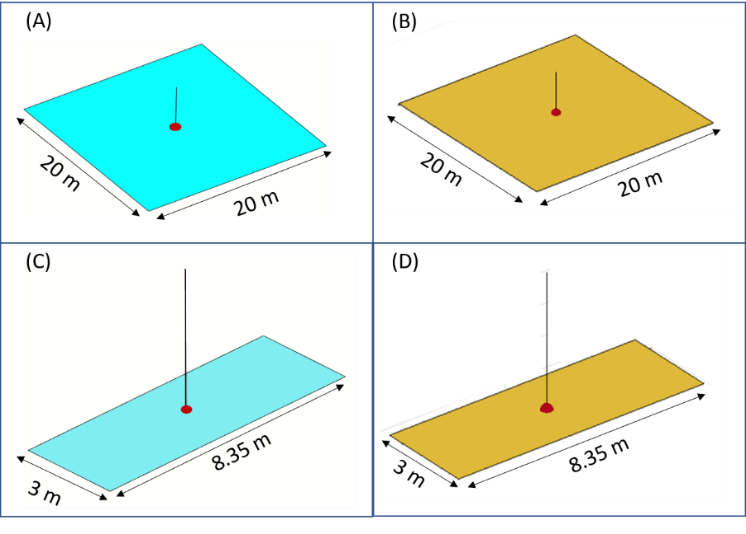


**Supplementary Figure 1**. Verification of the FDTD antenna model (A-C) by comparison with the MoM model (B‑D), in the two simulated conditions.

Frequency response of each model was obtained in the range 2 MHz – 30 MHz and compared, as shown in Supp. Fig. 2. A good agreement between the results obtained from the two methods is visible for both the real (resistance, blue curve) and the imaginary (reactance, red curve) part of the impedance, and for both the two grounds. Considering the 20 x 20 ground, both the software Sim4Life (s4l, FDTD) and the Antenna ToolBox (ATB, MoM) estimated the resonance frequency of the antenna at 16.6 MHz. At 16 MHz, values of resistance (R) and reactance (X) were 30 Ω and -19.86 Ω in s4l and 28.4 Ω and -32.8 Ω in ATB. With the smaller ground, the resonance frequency was computed at 17.06 MHz by both methods, and at 16 MHz, R_s4l_ =17 Ω and X_s4l_ = -33.4 Ω, while R_ATB_ =16.6 Ω e X_ATB_ = -45.9 Ω


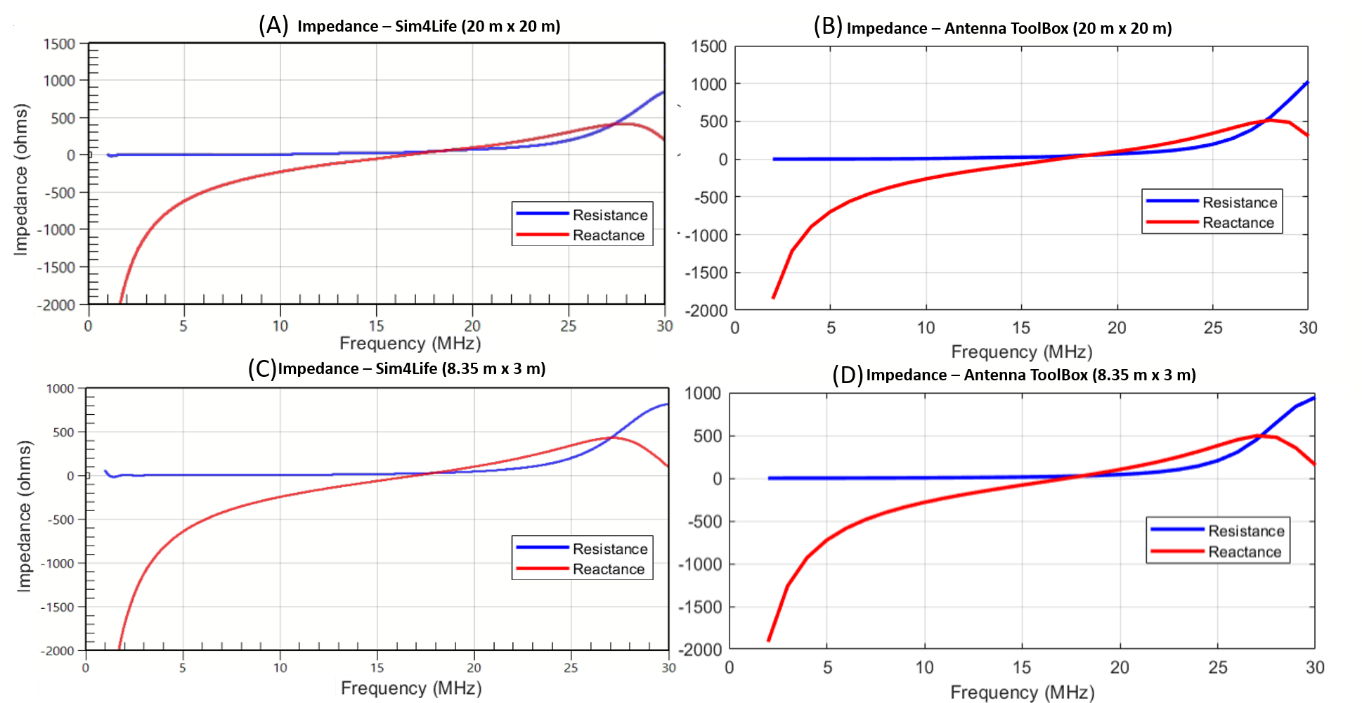


**Supplementary Figure 2**. Verification of the FDTD antenna model, comparison of antenna impedance: (A-B) monopole over 20 m x 20 m ground simulated in Sim4Life and in Antenna ToolBox. (C-D) monopole over 8.5 m x 3 m ground simulated in Sim4Life and in Antenna ToolBox.

Supp. Fig. 3 shows the 3D radiation diagram of the antenna computed with the two software packages, at 16 MHz for the two evaluated grounds. Maximum value of the directivity was estimated as D_s4l_ = 2.97 dB_i_ and D_ATB_ = 3.08 dB_i_ for the 20 m × 20 m ground, and D_s4l_ = 1.65 dB_i_ and D_ATB_ = 1.63 dB_i_ for the 8.35 m × 3 m ground.

Supp. Fig. 4 shows directivity along the the elevation (θ = 90°) and the azimuth (φ = 90°) computed with antenna placed over the two types of grounds by Sim4Life (A-C) and by the Antenna ToolBx (B‑D). A good agreement between the diagrams is visible, by comparing results in (A) with results in (B), and results in (C) with results in (D).

In conclusion, differences between the two solvers are up to 4% when estimating the directivity and 5% when estimating the resistance of the monopole. The resonance frequency was estimated as the same with both the methods.


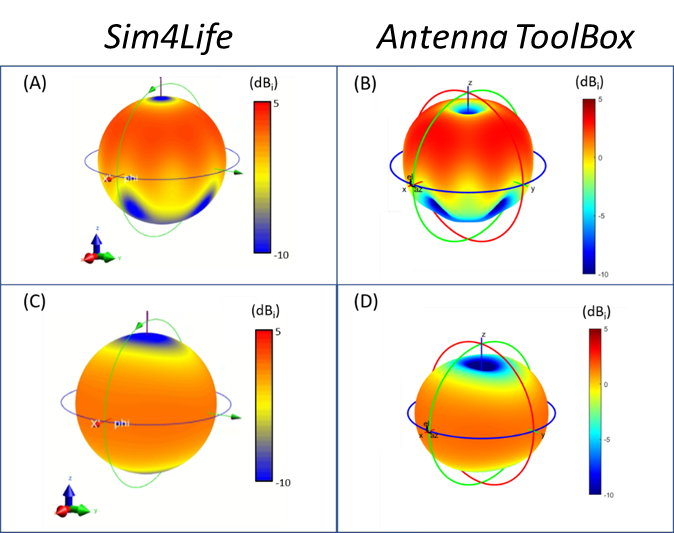


**Supplementary Figure 3**. Verification of the FDTD antenna model, comparison of antenna 3D radiation diagram (directivity of the antenna): (A-B) monopole over 20 m x 20 m ground simulated in Sim4Life and in Antenna ToolBox. (C-D) monopole over 8.5 m x 3 m ground simulated in Sim4Life and in Antenna ToolBox.


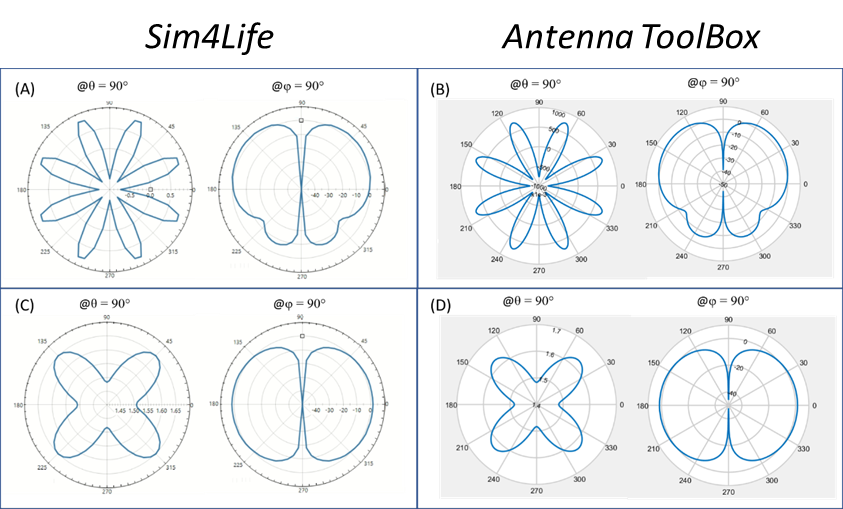


**Supplementary Figure 4**. Verification of the FDTD antenna model, comparison of antenna radiation diagram (directivity of the antenna) along the elevation (θ = 90°) and the azimuth (φ = 90°): (A-B) monopole over 20 m x 20 m ground simulated in Sim4Life and in Antenna ToolBox. (C-D) monopole over 8.5 m x 3 m ground simulated in Sim4Life and in Antenna ToolBox.

# Exposure scenario *b1*: vehicle with open manhole in presence of the operator touching the turret

Scenario b was further investigated with the human body leaning towards the edge of the manhole, with one arm bent and laid on the surface of the turret as shown in Supp. Fig. 5 (Scenario b1). The different posture was obtained with the “Poser” tool embedded in Sim4Life, that allows to manipulate the mesh of the human body models taken from the Posable version of the Virtual Population (ViP v.3). The characteristics of the radiating antenna and the simulation set up remained unchanged with respect to those applied for Scenario b and were described in the main article. To evaluate the effect of the contact with the manhole, the averaged whole body SAR (SARwb) and the peak of the SAR averaged over 10 g of tissue in the head (peak SAR10Avg) were computed and compared with results in Scenario b (Supp. Table 1). In the new position with the body in contact with the edge of the manhole and the turret, an increase in the SAR_wb_ is obtained, from 0.21 mW/kg to 0.52 mW/kg. Nevertheless the level induced is always well below the ICNIRP limit of 0.4 W/kg (i.e., 400 mW/kg). Conversely, a decrease in the peak SAR_10Avg_ of 26 % was found in the head. This was expected since in Scenario b1 the head was further from the antenna than it was in Scenario b. Furthermore we also calculated the limb peak SAR_10Avg_ over the bent arm, to evaluate whether the compliance with the limit imposed on the limbs was respected despite the contact with the turret. It resulted to be equal to 2.9 mW/kg, well below the 20 W/kg reported in the guidelines. Thus it can be concluded that for the radiating conditions simulated in this work, the guideline limits would be respected even with the human body in contact with the manhole.

**
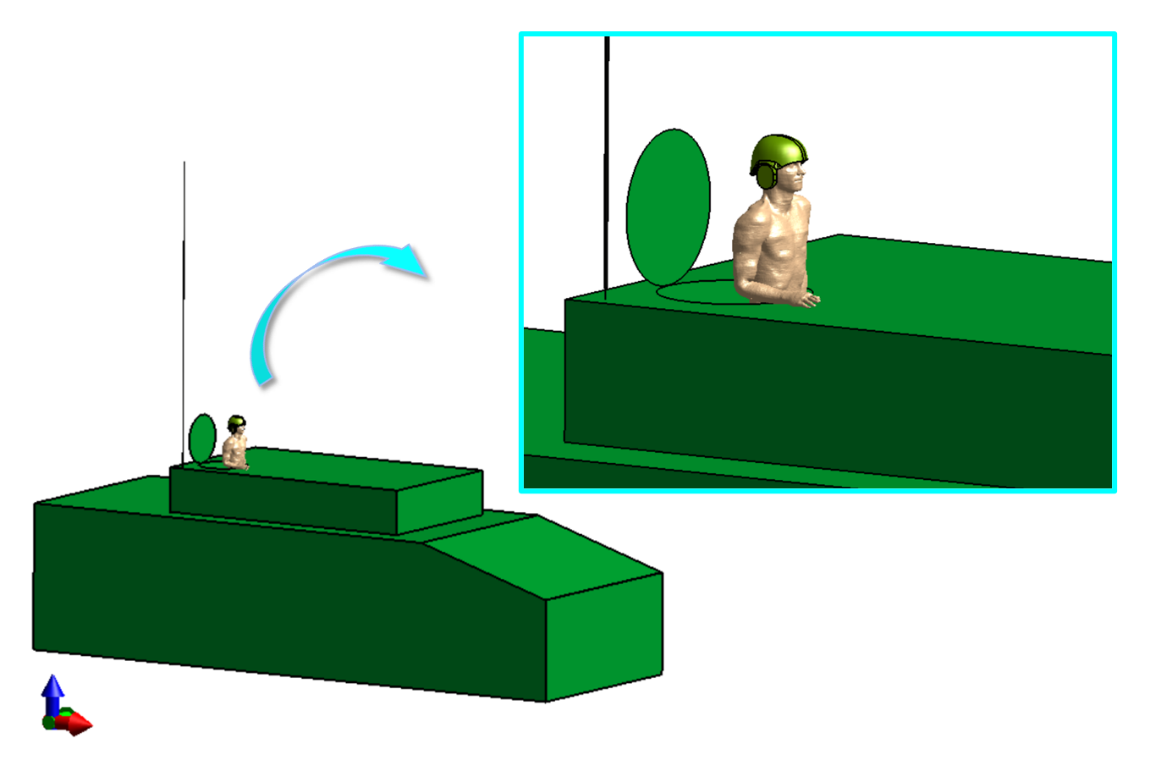
**

Supplementary Figure 5. Scenario b1: Model of a common scenario with the human body placed in proximity of the manhole edge and the arm bent over the turret.

Supplementary Table 1. Comparison between the estimated SAR values in exposure scenario b (described in the main body of the manuscript) and Scenario b1.

|  | *Scenario b* | *Scenario b_1_* | **Guidelines Limit** (1,3,4) |
| --- | --- | --- | --- |
| SAR_wb_ | 0.21 × 10^-3^ W/kg | 0.52 × 10^-3^ W/kg | 0.4 W/kg |
| peak SAR_10Avg_ | 4.2 × 10^-3^ W/kg | 3.1 × 10^-3^ W/kg | 10 W/kg |
